# The Cortical Dynamics of Dual-Task Standing in Older Adults

**DOI:** 10.1093/geroni/igab046.276

**Published:** 2021-12-17

**Authors:** Melike Kahya, On-Yee Lo, Junhong Zhou, Alvaro Pascual-Leone, Lewis Lipsitz, Jeffrey Hausdorff, Christoph Michel, Brad Manor

**Affiliations:** 1 Marcus Institute for Aging Research, Harvard Medical School, Boston, Massachusetts, United States; 2 Hebrew SeniorLife/Harvard Medical School, Boston, Massachusetts, United States; 3 Harvard Medical School/Hebrew SeniorLife, Roslindale, Massachusetts, United States; 4 Harvard Medical School, Boston, Massachusetts, United States; 5 Hebrew SeniorLife, Boston, Massachusetts, United States; 6 Tel Aviv University, Tel Aviv University, Tel Aviv, Israel; 7 University of Geneva, University of Geneva, Geneve, Switzerland; 8 Hinda and Arthur Marcus Institute for Aging Research, Harvard Medical School, Boston, Massachusetts, United States

## Abstract

In older adults, the extent to which performing a cognitive task when standing diminishes postural control is predictive of future falls and cognitive decline. The cortical control of such “dual-tasking,” however, remains poorly understood. Electroencephalogram (EEG) studies have demonstrated that the level of attention and cognitive inhibitory activity during cognitive task performance can be quantified by changes in brain activity in specific frequency bands; namely, an increase in theta/beta ratio and a decrease in alpha-band power, respectively. We hypothesized that in older adults, dual-tasking would increase theta/beta ratio and decrease alpha-band power, and, that greater alpha-band power during quiet standing would predict worse dual-task performance. To test this hypothesis, we recorded postural sway and EEG (32-channels) in 30 older adults without overt disease as they completed trials of standing, with and without verbalized serial subtractions, on four separate visits. Postural sway speed, as well as absolute theta/beta power ratio and alpha-band power, were calculated. The theta/beta power ratio and alpha-band power demonstrated high test-retest reliability during quiet and dual-task standing across visits (intra-class correlation coefficients >0.70). Compared with quiet standing, dual-tasking increased theta/beta power ratio (p<0.0001) and decreased alpha-band power (p=0.002). Participants who exhibited greater alpha-band power during quiet standing demonstrated a greater dual-task cost (i.e., percent increase, indicative of worse performance) to postural sway speed (r=0.3, p=0.01). These results suggest that in older adults, dual-tasking while standing increases EEG-derived metrics related to attention, and, that greater cognitive inhibitory activity during quiet standing is associated with worse dual-task standing performance.

